# Forest Fire Detection Based on Improved YOLO11

**DOI:** 10.3390/s26103094

**Published:** 2026-05-14

**Authors:** Jialong Gao, Yanqiao Zhao, Bowen Chen

**Affiliations:** School of Measurement and Control Technology and Communication Engineering, Harbin University of Science and Technology, Harbin 150080, China; 2320610169@stu.hrbust.edu.cn (J.G.); 2420610197@stu.hrbust.edu.cn (B.C.)

**Keywords:** forest fire detection, YOLO11, lightweight network, ShuffleNetV1, SPD-Conv

## Abstract

Addressing the limitations of inadequate model lightweighting and suboptimal detection accuracy in forest fire detection systems, a refined forest fire detection approach based on an improved YOLO11 architecture is proposed. Based on the YOLO11 network architecture, the backbone network is modified by integrating the ShuffleNetV1 module to achieve efficient and lightweight model deployment. Additionally, the incorporation of the SPD-Conv convolutional module not only expands the receptive field to strengthen the aggregation of semantic features for large-scale flame targets but also precisely preserves fine-grained edge and texture information of small-scale smoke targets. The experimental results show that the improved model achieves a real-time inference speed of 148.3 FPS, a 22.5% reduction in parameter count, a 0.3% improvement in mAP, and a 15.0% decrease in GFLOPs. It achieves the lightweight design of the improved YOLO11 and the improvement of detection accuracy for forest fire targets.

## 1. Introduction

Forest fires constitute one of the most pressing environmental crises exacerbated by anthropogenic climate change [1]. As global average temperatures rise persistently, drought stress has intensified across vast terrestrial regions, reducing vegetation moisture content and increasing the likelihood of wildfire ignition events [2,3]. Concurrently, wildfire emissions release large quantities of carbon dioxide (CO_2_), which in turn amplifies the feedback loops of anthropogenic climate change [4]. Therefore, timely detection of fire risks and prediction of fire spread at the early stage of a fire are of great significance [5]. Such early detection capability not only minimizes the ecological and socioeconomic damage caused by wildfires but also fundamentally curbs the potential for large-scale wildfire spread.

Before the widespread adoption of deep learning, forest fire monitoring primarily relied on recognizing fire targets in images based on flame color characteristics, shape features, motion properties, or texture information [6]. To date, deep learning has been extensively integrated into object detection algorithms. Based on the procedural steps and task decomposition strategies of object detection, such algorithms are classified into one-stage and two-stage paradigms. The first category is two-stage object detection, exemplified by R-CNN [7], Fast R-CNN [8], and Faster R-CNN [9]. Such algorithms decompose the object detection task into two sequential stages: region proposal and region classification. While this yields superior recognition accuracy, such methods suffer from relatively low detection speed, a large model memory footprint, and high computational demands, making them unsuitable for forest fire detection scenarios. The second category is one-stage object detection, exemplified by YOLO [10] and SSD [11]. Such approaches directly predict the bounding box and category probability of target objects, obviating the need for an independent region proposal stage. Consequently, such methods exhibit a relatively simple architecture and high detection speed, thereby emerging as a research hotspot in the field of forest fire image detection. Furthermore, owing to its excellent applicability, real-time performance, and enhanced robustness to interference from complex backgrounds, YOLO is particularly well-suited for forest fire detection tasks. With the advancement of technology, YOLO has subsequently evolved into enhanced variants, including YOLOv2 [12], YOLOv3 [13], YOLOv4 [14], and YOLOv5 [15]. However, these mainstream YOLO variants still exhibit inherent limitations in the detection of small forest fire targets.

In 2018, Wu et al. [16] conducted a comparative analysis of the SSD, Faster R-CNN, and YOLO algorithms for forest fire detection. Their findings demonstrated that YOLO-series algorithms exhibited a marked superiority over other detection models in terms of detection speed. In 2019, Ren et al. [17] from Zhejiang Sci-Tech University constructed a multi-scenario fire database and improved the small-target detection performance based on the YOLOv3 framework. They optimized the localization accuracy of flame and smoke targets via multi-scale feature fusion, adjusted the network architecture to meet real-time operational requirements, and achieved an average detection speed of 30 FPS under complex lighting conditions. While representing a typical early application of YOLO series algorithms in forest fire detection, this work still leaves considerable room for improvement in detection accuracy. In 2022, the FireNet team [18] proposed a forest fire detection model in Sensors. Trained on Landsat-8 imagery, this model integrated YOLOv5 with U-Net to detect active fires and burning combustible materials. Innovatively, to address the irregular morphologies of flame targets, it adopted non-rectangular multi-bounding boxes for the detailed annotation of flame contours. Nevertheless, this model was exclusively designed for flame detection and failed to realize forest fire detection for early-stage smoke targets. In 2022, Gao et al. [19] from Nanjing Forestry University proposed a forest fire detection method based on an improved YOLOv5 architecture. To address the challenges posed by complex backgrounds and numerous small targets in unmanned aerial vehicle (UAV) aerial imagery, the NAM attention mechanism was integrated to normalize pixel weights and suppress interference from non-salient features. Concurrently, the ObjectBox detector was adopted to optimize multi-scale object detection; it was further combined with Mosaic data augmentation and the CIoU loss function to improve localization accuracy. The experimental results indicated that the improved model achieved a 3% improvement in average precision on a self-constructed dataset, significantly outperforming the original YOLOv5 model; however, this model exhibited poor generalization capability owing to the limited scale of the dataset. In 2024, Wu et al. [20] from Jilin Agricultural University developed the Dynamic Snake Convolution (DysConv) and Global Attention Mechanism (GAM) modules based on the YOLOv8 model. Tailored for complex scenarios such as diurnal variation and fog interference, these modules enhance the feature extraction capability for smoke and fire targets. They optimized anchor box localization via the WIoU loss function and integrated the DyHead module to enhance the adaptability of the detection head to spatial dimensions and task-aware capabilities. These modifications to the attention mechanism and loss function yielded promising results; however, the resultant model exhibited poor lightweight performance. In 2025, Ma et al. [21] proposed a lightweight real-time forest fire detection method designated RF-YOLOv8s. This method prioritizes spatial features of the receptive field to enhance the feature learning capability for flame targets and adopts Inner-CIoU as a novel bounding box loss function. By regulating auxiliary bounding boxes, it alleviates the poor generalization of bounding box regression and accelerates the convergence speed of the model. Nevertheless, the model’s robustness, detection capability in complex scenarios, and lightweight performance still leave considerable room for further optimization. While unmanned aerial vehicle (UAV)-based object detection has gained escalating significance in forest fire surveillance, a multitude of critical challenges persist in this field. In 2026, H. Zhang et al. [22]. targeted the long-standing challenge of inaccurate detection of early-stage fire points (i.e., small-scale flames) in aerial imagery. From the UAV imaging perspective, the authors developed a novel YOLOv8s-MS model integrated with multi-scale feature fusion and attention residual mechanisms. Specifically, this model incorporates a multi-scale feature fusion (MSFF) strategy, incorporates an additional shallow detection head, and eliminates the deep-level detection head. Subsequently, a custom residual module grounded in the SKAttention mechanism is further devised for feature refinement. The experimental results demonstrate that the proposed method yields substantial performance enhancements in flame target detection tasks, achieving mean average precision (mAP) values of 81.8% and 95.7%, respectively.

The remainder of this paper is organized as follows: Section 2 introduces the original YOLO11 model and the innovations proposed in this paper; Section 3 describes the experimental preparations and analyzes the experimental results; finally, Section 4 presents the main conclusions, as well as the limitations of the method and directions for future work.

## 2. Method

### 2.1. YOLO11 Network Architecture

YOLO11, a next-generation computer vision model developed by Ultralytics, incorporates architectural advancements over its predecessors, including the integration of the C3K2 module into the backbone network and the incorporation of the C2PSA attention module after the SPPF layer. This model has demonstrated superior performance in the field of object detection [23,24]. When applied to forest fire detection tasks, the backbone and neck structures of YOLO11 demonstrate enhanced feature extraction capabilities for small flame targets. Furthermore, YOLO11 adopts dynamic anchor boxes integrated with adaptive task-aligned learning, thereby enhancing its adaptability for localizing irregular forest fire targets. Compared with its predecessors, YOLO11 also boasts notable advantages in inference speed and model compactness. Consequently, this study adopts YOLO11 as the baseline model for forest fire detection tasks. Nevertheless, with the growing adoption of portable mobile devices (e.g., unmanned aerial vehicles, UAVs) in forest fire detection tasks, deploying the algorithm on such platforms requires further optimizations of YOLO11 in terms of lightweight design and detection accuracy.

**Figure 1:**
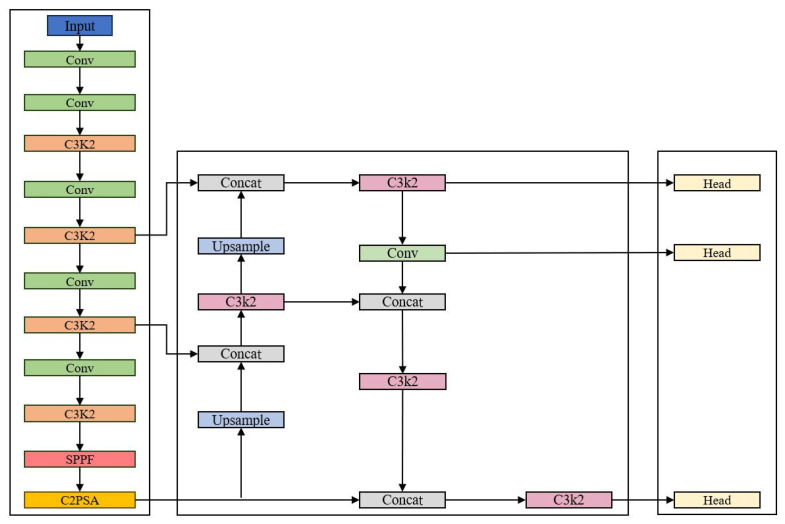
YOLO11 model architecture.

The architecture of YOLO11 is primarily composed of three core components: a backbone network, a neck network, and a detection head [25]. The backbone network incorporates the C3K2 module, which replaces conventional large-kernel convolutions with multiple 3 × 3 small convolutions. This design reduces computational overhead while maintaining robust feature extraction capabilities. The neck network innovatively integrates the lightweight C3K2 convolutional module and the C2PSA dual-branch partial spatial attention module into the multi-scale feature fusion pipeline. This structure not only reduces computational costs via small convolutions but also enhances the perception of critical regions through targeted attention mechanisms, making it highly suitable for small-object detection. The detection head employs depthwise separable convolutions, which substantially reduce both the number of model parameters and the overall computational complexity. Overall, YOLO11 achieves effective model lightweighting while maintaining superior real-time detection performance, rendering it well-suited for high-efficiency object detection tasks on edge devices and mobile platforms. The overall architecture of the YOLO11 model is illustrated in Figure 1.

When YOLO11 is applied to forest fire detection, the C3K2 module, in comparison with other YOLO variants, retains the residual structure of the C3 module. It achieves multi-level feature fusion via branch convolutions and residual connections. Concurrently, it leverages residual paths to mitigate gradient vanishing in deep neural networks, thereby ensuring the backbone network’s ability to extract features of forest fires. Furthermore, the C3K2 module adopts grouped convolution and a bottleneck structure, which substantially reduces the number of parameters and computational overhead relative to conventional convolutional modules. Nevertheless, despite the lightweight design of the C3K2 module, in edge deployment scenarios for forest fire detection, it still results in excessive parameters and computational overhead. This increases inference latency and hinders the rapid identification of early-stage fire incidents.

In traditional convolutional neural network (CNN) architectures, strided convolutions and pooling layers within the neck network collaboratively execute the core process of “downsampling—feature enhancement—multi-scale construction.” This jointly facilitates YOLO11 in achieving multi-scale detection and optimizing localization accuracy for forest fire targets. Nevertheless, for edge deployment adaptation, strided convolutions fail to discriminate between “target feature channels” and “background feature channels” during dimension compression. Moreover, the indiscriminate aggregation by pooling layers further impairs the integrity of feature representation. Ultimately, this results in an imbalance between “lightweighting” and “detection accuracy,” leading the model to fail to simultaneously satisfy the computational constraints of edge deployment and the accuracy requirements for forest fire detection.

### 2.2. YOLO11 Algorithm Improvement

To mitigate the aforementioned limitations, this study presents a modified network architecture based on YOLO11 that preserves detection accuracy while simultaneously enabling model lightweighting.

#### 2.2.1. Incorporating the ShuffleNetV1 Module

To address the challenge of excessive parameter counts and high computational overhead introduced by the C3K2 module in YOLO11-based forest fire detection models—limitations that hinder deployment on resource-constrained edge devices—this study replaces all C3K2 modules in the backbone network of the baseline model with lightweight ShuffleNetV1 [26] units.

ShuffleNetV1 is architected around the core principles of grouped convolution and channel shuffle. Leveraging sparse connectivity and an efficient feature interaction mechanism, this module mitigates the inherent limitations of the C3K2 module while enhancing the model’s scene adaptability for forest fire detection tasks.

The core advantages of ShuffleNetV1 arise from the synergistic integration of grouped convolution and channel shuffle. This design paradigm not only enables model lightweighting but also enhances the efficiency of feature interaction and aggregation. Grouped convolution facilitates the fine-grained extraction of intra-group features, enabling the precise capture of weak local features from ultra-small targets. In contrast, channel shuffle alleviates channel-wise isolation, facilitating the fusion of multi-dimensional features and consequently reducing the miss detection rate substantially. The specific functionalities and underlying mechanisms of these two core components are elaborated as follows:

(1) Group Convolution

Grouped convolution partitions the channels of an input feature map into multiple disjoint groups, where each group undergoes independent convolution operations. Its primary functionalities include computational cost reduction—whereby the distribution of convolution operations across multiple groups yields a substantial reduction in computational overhead—and parameter efficiency enhancement, as each group employs dedicated convolution kernels, thereby reducing the total number of trainable parameters.

(2) Channel Shuffle

Channel shuffle is an auxiliary operation designed to enhance inter-channel feature interaction and is typically deployed in tandem with grouped convolution. While grouped convolution effectively reduces computational overhead, it induces inter-group information segregation, which in turn impairs feature representational capacity. In response, channel shuffle rearranges the channel dimensions of feature maps, facilitating information exchange across different groups and thus augmenting feature representational capacity.

**Figure 2:**
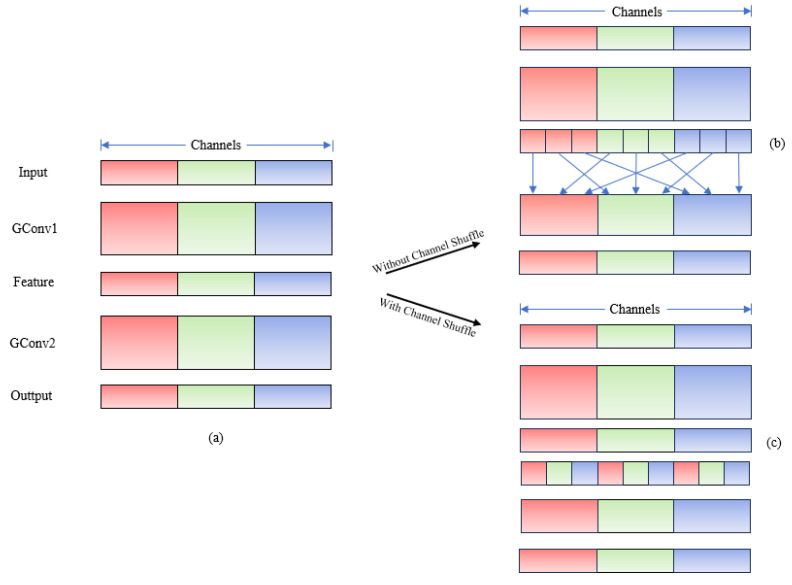
Channel shuffle operation flow. Where (**a**) is stacked convolutional layers; (**b**) is Without channel shuffle. and (**c**) is With channel shuffle.

Subsequent to grouped convolution, the channel shuffle mechanism randomly permutes the channel dimensions of feature maps along the channel axis. This operation effectively mitigates the channel information fragmentation issue induced by grouped convolution, while preserving the model’s lightweight properties to align with the edge deployment requirements of forest fire detection systems. The implementation of the channel shuffle mechanism within the ShuffleNet [27] architecture is illustrated in Figure 2.

In the aforementioned figure, Panel (a) depicts two stacked convolutional layers with an identical number of groups, where each output channel exhibits connectivity exclusively to input channels within the same group. Panel (b) illustrates the complete correlation between input and output channels when Group Convolution 2 (GCov2) aggregates features from distinct groups subsequent to Group Convolution 1 (GCov1), in the absence of channel shuffle. Panel (c) presents an identical implementation to that in Panel (b) but integrates channel shuffle to enable cross-group information exchange, thereby facilitating more efficient and robust feature learning across the network.

**Figure 3:**
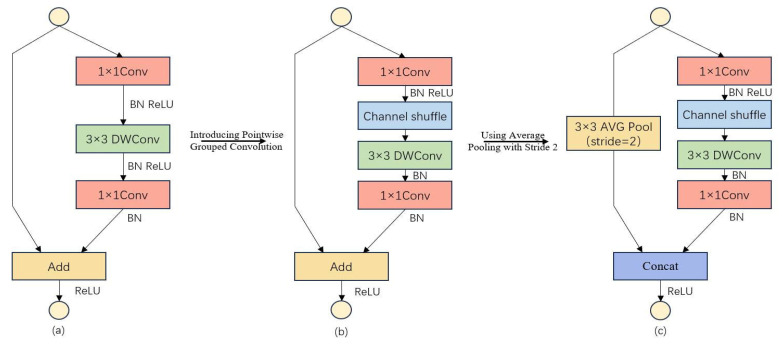
Basic module of the ShuffleNet architecture. Where (**a**) is epthwise separable convolution fused with element-wise Add; (**b**) is Embedded grouped point-wise convolution. and (**c**) Introduced a dedicated downsampling unit.

The fundamental building block of the ShuffleNet architecture serves as the definitive modular carrier for the synergistic operation of these two core mechanisms. To fully exploit the lightweighting benefit of grouped convolution and the inter-channel feature interaction capability of channel shuffle, ShuffleNetV1 incorporates a standardized fundamental module structure, enabling seamless integration and efficient coordination between the two mechanisms. The detailed architecture of this module is illustrated in Figure 3.

In the presented figure, Panel (a) depicts a fundamental bottleneck unit that leverages depthwise separable convolution (DWConv) and element-wise addition (Add) for feature fusion. Panel (b) illustrates the integration of pointwise grouped convolution (GCo) and channel shuffle operations into the standard bottleneck unit, a design modification intended to enhance feature representational capacity. Panel (c) shows a ShuffleNet unit optimized for spatial downsampling, which employs stride-2 average pooling (AVG Pool) and depthwise separable convolution. Subsequent to these operations, features undergo further refinement via channel shuffle and pointwise grouped convolution, with feature aggregation ultimately achieved through a concatenation operation (Concat).

Consequently, the replacement of C3K2 modules with ShuffleNetV1 units in the backbone network of the YOLO11 model yields multifaceted performance gains: it reduces computational overhead and parameter volume, decreases memory consumption, enhances inter-group feature interaction, improves feature representational capacity, further mitigates computational complexity, boosts inference speed, and reduces the miss detection rate. These optimizations render the modified model better suited for deployment on resource-constrained edge devices for forest fire detection tasks.

#### 2.2.2. Incorporating the SPD-Conv Convolutional Module

For YOLO11-based forest fire detection algorithms, a critical limitation emerges whereby strided convolutions and pooling layers induce irreversible feature loss for small forest fire targets. This shortcoming not only hinders compliance with high-precision detection requirements but also exacerbates the trade-off between model lightweighting and detection accuracy in the resulting framework. To address this challenge, this study replaces the strided convolutions and pooling layers within the network with the SPD-Conv convolutional module [28], which comprises a non-strided convolution layer and a space-to-depth transformation layer.

**Figure 4:**
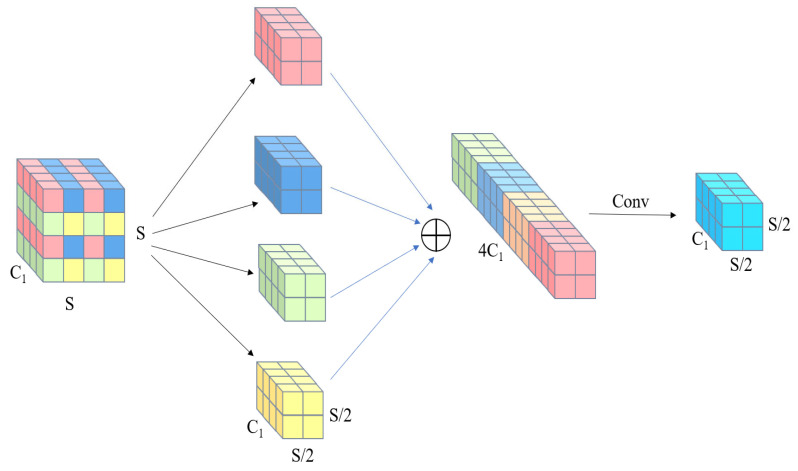
Structure of the SPD convolution neural network.

The SPD-Conv module executes downsampling on feature maps via the space-to-depth layer while preserving all channel-wise information throughout the process. For an arbitrary intermediate feature map X, the space-to-depth layer partitions X into a set of sub-feature maps according to a predefined scaling ratio, where each sub-feature map implements downsampling of the original feature map X. These sub-feature maps are then concatenated along the channel dimension to generate a new feature map X’. As the spatial size of feature map X’ are reduced, its channel dimension increases by the square of the spatial downsampling factor.. Subsequent to the space-to-depth transformation, a non-strided convolution layer is incorporated to transform X’, thereby preserving the maximum amount of discriminative feature information [12]. The architectural design of the SPD-Conv module is illustrated in Figure 4.

In this context, depicts a conventional feature map with a channel dimension of C1 and spatial dimensions (height, width). Via the space-to-depth transformation, spatial pixel blocks are rearranged into the channel dimension, expanding the channel count to 4C1 while downsampling the spatial dimensions by a factor of 2. Subsequent to this, distinct channel groups are concatenated along the channel axis. The aggregated feature map may then undergo element-wise addition with other processed feature maps. Finally, a stride-1 convolution is applied to the resultant feature map, reducing the channel dimension to 2 while preserving the spatial resolution—this resolution remains at one-half of the original feature map’s spatial size.

Within the SPD-Conv framework, subsequent to the SPD layer remapping spatial information from the input feature map to the channel dimension, the non-strided convolution layer (stride = 1) is tasked with processing these rearranged feature maps. Given a stride of 1, the convolution operation preserves spatial resolution, thereby retaining fine-grained detail information while reducing the channel dimension. This design paradigm significantly enhances feature representational capacity, making it particularly well-suited for processing small-target imagery—where critical details are prone to loss in conventional convolutional neural networks (CNNs). The non-strided convolution layer employs a stride-1 operation, whereby the convolution kernel traverses the input feature map in a pixel-wise manner, ensuring full spatial coverage. This design maximizes information retention and yields rich, discriminative feature representations. The non-strided convolution layer serves as a critical downstream component of the space-to-depth (SPD) layer. Through this integrated design, the network can capture fine-grained details more effectively, thereby boosting model performance on complex vision tasks.

Consequently, the replacement of strided convolutions and pooling layers with the SPD-Conv module within the network—while incurring a modest increase in computational overhead—still satisfies the real-time detection requirements of forest fire monitoring systems. Concurrently, this modification substantially enhances the model’s robustness in complex scenarios and reduces the miss detection rate. Ultimately, this yields synergistic optimization defined by “controllable lightweighting overhead and leapfrog improvements in detection accuracy”.

**Figure 5:**
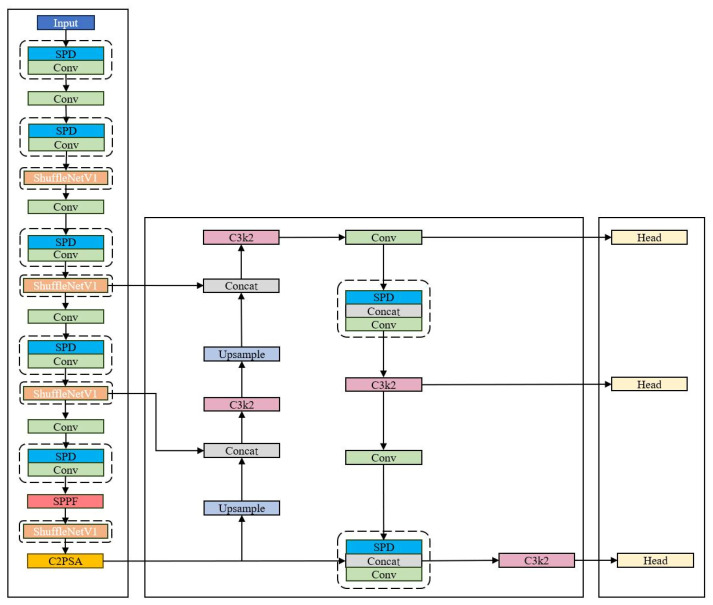
The improved YOLO11 model architecture.

Building on the aforementioned modifications, the network architecture of the improved YOLO11 forest fire detection algorithm is illustrated in Figure 5. Dashed boxes in the figure denote the modified components. The enhanced model preserves the full detection head architecture of the baseline YOLO11 model to maintain inference efficiency while achieving simultaneous improvements in model lightweighting and detection accuracy via structural optimization of the backbone and neck networks.

As shown in Figure 5 above, the ShuffleNetv1 and SPD-Conv modules are integrated into the YOLO11 network to achieve a better balance between lightweight design and detection accuracy of the forest fire detection model, and the effectiveness of the introduced modules is verified through the following experiments.

## 3. Experiment and Result Analysis

### 3.1. Dataset Construction

Within the context of model training, the representativeness, diversity, and volume of dataset samples exert a critical influence on the training process of detection models. To meet the requirements of model training, an extensive forest fire dataset was curated to serve as the training and validation subsets for the proposed model. The public datasets utilized in this study are illustrated in Table 1.

However, public datasets suffer from a scarcity of samples depicting small-target fire smoke and early-stage fire events, which impairs the ability of detection models to achieve accurate early-fire detection and thus fails to satisfy the early-warning requirements of forest fire prevention and control systems. To address this limitation, this study supplements public datasets with a custom-curated dataset, which mitigates the inherent deficiencies of existing public datasets and thus ensures the validity and reliability of the model training process. The custom-curated dataset comprises pan–tilt–zoom (PTZ) forestry monitoring videos provided by the Heilongjiang Academy of Forestry, as well as aerial forestry videos captured via unmanned aerial vehicles (UAVs) during routine forestry operations. For the collected forest fire monitoring video clips, frame sampling was conducted at a rate of 16 frames per second to select valid images that depict critical scenarios, including small flame regions and concurrent smoke–flame occurrences.

When employing the aforementioned data acquisition pipelines, several issues may arise, including corrupted image files, duplicate entries, and inconsistencies in image resolution and file format. To identify corrupted files, we validate three key attributes: zero-byte file sizes, anomalous storage allocation, and consistency between the file extension and its actual MIME type. To eliminate duplicates, we traverse the directory structure using Python’s os.walk and compute perceptual hashes (pHash) for each image. Images are flagged as duplicates if their Hamming distance is ≤5, or as identical copies if their MD5 hashes are exactly matched. For format standardization, the JPEG (.jpg) format was adopted as the universal standard, balancing trade-offs between model I/O efficiency, flame feature preservation, and storage overhead. Following the removal of corrupted, duplicate, and non-conforming images, a consistent file naming convention was enforced across the entire dataset using the Python Imaging Library (PIL/Pillow).

Subsequent to a series of preprocessing steps—including data annotation, data filtering, and image standardization—a forest fire detection image dataset was constructed to serve as the training and validation corpus for the proposed model. This study constructs the final experimental dataset by fusing the aforementioned public datasets with the custom-curated dataset described above. The final dataset utilized in this experiment comprises a total of 5000 representative images, which were selected from the aforementioned public datasets and the custom-curated dataset.

Specifically, public datasets comprise 70% of the total set (3500 images). To mitigate the limited scene diversity and geographical coverage of existing public datasets, self-collected data contribute the remaining 30% (1500 images). All images are required to maintain sufficient resolution and visual clarity, such that fire-related features are not obscured by low-quality sampling. Severe blur or distortion is excluded to guarantee reliable feature extraction by the improved YOLO11 model. Furthermore, the dataset spans the full fire life cycle, including incipient fires, developing fires, extinguished fires, and non-fire backgrounds. It also incorporates images captured under diverse temporal conditions—daytime, nighttime, and dusk—thereby establishing a high-quality benchmark to support subsequent experimental validation.

### 3.2. Experimental Environment and Parameter Configuration

All experiments conducted in this study were performed under the hardware and software configurations detailed in Table 2.

During the model training phase, to standardize input images, all images in the dataset were uniformly resized to a resolution of 640 × 640 pixels using the cv2.resize() function from the OpenCV library. The training parameters were set as follows: batch size = 8, total training epochs = 200, initial learning rate = 0.01, momentum coefficient = 0.937, and weight decay coefficient = 0.0005. To balance training sufficiency and overfitting risk, the core validation metric (mAP@0.5) was continuously monitored throughout the training process. If this metric failed to improve or exhibited a continuous decline over successive epochs, an early stopping mechanism was triggered to terminate training, and the optimal model weights corresponding to the peak value of this metric were saved.

### 3.3. Experimental Evaluation Metrics

Given that forest fire detection constitutes a typical binary classification task—where fire-related targets are designated as the positive class and all other interfering elements as the negative class—this study employs recall (R), precision (P), average precision (AP), and mean average precision (mAP) as core metrics to quantify the algorithm’s detection performance. Recall (R) and precision (P) are fundamental evaluation metrics in information retrieval, traditionally employed to assess the performance of retrieval systems. Precision (P) quantifies the proportion of true positive predictions among all positive predictions, while recall (R) measures the fraction of actual positive samples that are correctly identified. Average precision (AP) encapsulates the overall recognition accuracy of a model across different confidence thresholds, while mean average precision (mAP) denotes the mean value of AP scores across all target categories in the task. Concurrently, to assess computational overhead and detection complexity, computational load (GFLOPs, giga floating point operations per second) and inference frame rate (FPS, frames per second) are adopted as supplementary metrics to characterize model complexity. Among these metrics, mAP@0.5 is selected as the primary performance metric, where mAP@0.5 corresponds to the mAP value calculated with an intersection over union (IoU) threshold set to 0.5.

### 3.4. Module Ablation Experiment

To validate the efficacy of the proposed modules, this study performed an ablation study on the forest fire detection dataset described previously. All modules were trained under identical server hardware and software configurations, with the same dataset partition and training epoch count employed for consistency. During the inference phase, a unified image input resolution was adopted, and ablation tests were conducted incrementally on the ShuffleNetV1 and SPD-Conv modules to quantify the specific impact of each modified module on the model’s overall detection performance. The results of these ablation experiments are illustrated in Table 3.

Integrating the ShuffleNetv1 module into the backbone network of the YOLO11 model yields a 28.7% reduction in the number of parameters, a 16.3% drop in GFLOPs, and a mere 0.5% decline in mean average precision (mAP). Against the backdrop of the notable lightweighting gains for the model, this marginal mAP decline has a negligible effect on the overall model accuracy. Incorporating the SPD-Conv convolutional module into the network results in a modest increase in parameter count, a change that still satisfies the practical requirements for forest fire detection, alongside a 0.6% improvement in mAP. Co-integrating the ShuffleNetv1 module with space-to-depth layers and non-strided convolutional layers induces a 22.5% reduction in parameter count, a 15.0% decrease in GFLOPs, and a 0.3% rise in mAP. Simultaneous co-integration of the ShuffleNetv1 and SPD-Conv modules not only offsets the marginal accuracy decline of the model arising from the standalone integration of the ShuffleNetv1 module but also delivers a measurable improvement in accuracy relative to the original YOLO11 model. Furthermore, this synergy enables the model to achieve a more optimal trade-off between lightweighting and accuracy, thereby validating the practical efficacy of the proposed method for forest fire detection applications.

### 3.5. Model Comparison Experiment

To validate the efficacy of the proposed modified method for forest fire detection, this study selected five state-of-the-art object detection algorithms for comparative benchmarking, namely, YOLOv8, YOLOv9, YOLOv10, the baseline YOLO11 model, and Faster R-CNN. All comparative models were trained under identical experimental conditions, including the same dataset partition, hyperparameter configuration, and hardware/software environment. The results of these comparative benchmarking experiments are illustrated in Table 4.

As depicted in Table 4, the proposed improved algorithm outperformed all comparative algorithms, achieving a mean average precision (mAP) of 84.6%—a 1.0% relative improvement over the YOLOv8 model. In comparison with the YOLOv10 algorithm, the proposed method achieved a 2.4% improvement in detection accuracy, alongside a 30.0% reduction in network parameter count and a 27.7% decrease in GFLOPs (giga floating point operations per second). These experimental results collectively validate the efficacy of the integrated ShuffleNetV1 module and SPD-Conv module proposed in this study, which underpins the performance gains observed in the comparative experiments.

### 3.6. Visualization Analysis

**Figure 6:**
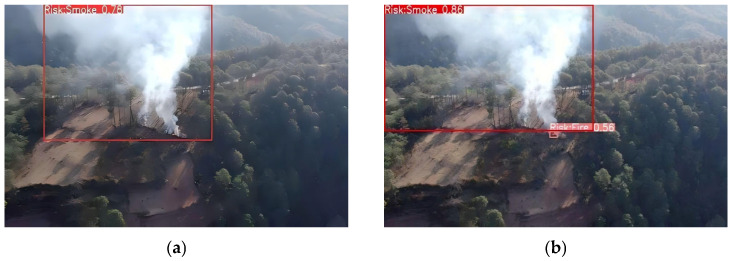
Comparison under long-distance scenes. Where (**a**) is the original YOLO11 and (**b**) is the improved YOLO11.

**Figure 7:**
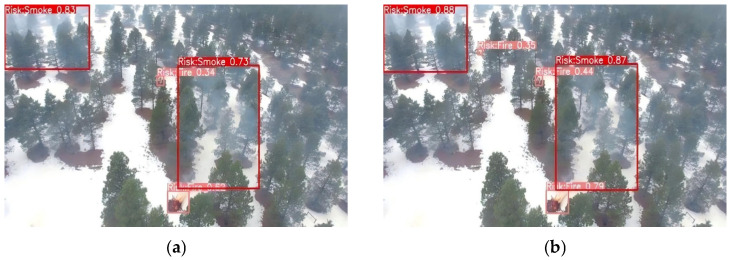
Comparison under low-smoke concentration scenes. Where (**a**) is the original YOLO11 and (**b**) is the improved YOLO11.

**Figure 8:**
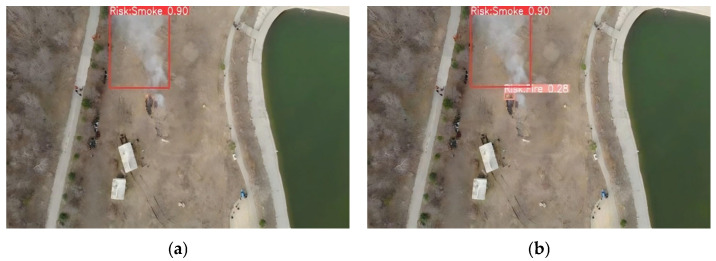
Comparison under small flame area scenes. Where (**a**) is the original YOLO11 and (**b**) is the improved YOLO11.

**Figure 9:**
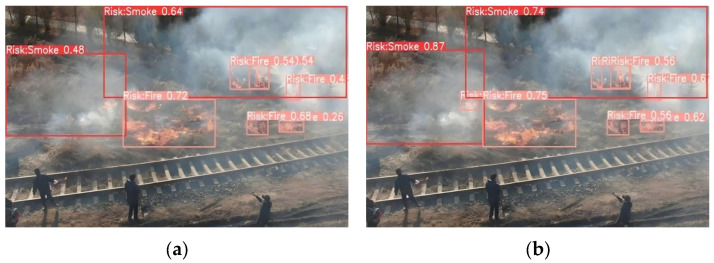
Comparison under scenes where smoke and flame coexist. Where (**a**) is the original YOLO11 and (**b**) is the improved YOLO11.

Ablation and comparative experiments provide only quantitative insights into model optimization and fail to characterize variations in model performance under real-world deployment scenarios. To address the limitations of the aforementioned quantitative experiments, and to more intuitively compare the anchor box prediction distributions before and after modification, as well as the efficacy of the improved model in real-world scenarios, we conducted inference on images from the dataset’s test set and selected a subset of representative images for qualitative visual analysis. These qualitative visual results more intuitively illustrate the discernible differences in detection performance between the baseline and improved models in real-world applications. The comparative detection results are illustrated in Figure 6, Figure 7, Figure 8 and Figure 9, which correspond to four typical forest fire scenarios: long-distance detection scenes, low smoke concentration scenes, small flame region scenes, and concurrent smoke–flame scenes, respectively.

For long-distance detection scenes, the baseline model failed to identify all smoke regions in the input images, exhibiting clear miss detection events. In contrast, the improved YOLO11 model successfully detected all smoke regions within the scene and yielded significantly higher confidence scores for the detected targets.

For scenes with low smoke concentration, the qualitative comparison results are presented in Figure 7. The baseline model failed to detect the low-concentration smoke regions in the scene. In contrast, the improved YOLO11 model successfully identified both the flame regions and the low-concentration smoke regions within the scene.

For scenes featuring small flame regions, the qualitative comparison results are illustrated in Figure 8. The baseline model only identified the left-side flame region and the right-side smoke region but failed to detect the smaller flame region located beneath the right-side smoke. In contrast, the improved YOLO11 model successfully identified not only the left-side flame region but also the smaller right-side flame region, in addition to detecting the right-side smoke region. Furthermore, relative to the baseline model, the improved YOLO11 model yielded significantly higher confidence scores for both smoke and flame detection outputs.

For concurrent smoke–flame scenes, the qualitative comparison results are illustrated in Figure 9. The baseline model only identified the upper smoke region, the flame region at the lower left of the smoke, and the flame region beneath the smoke, but failed to detect the flame region embedded within the smoke plumes. In contrast, the improved YOLO11 model successfully identified not only the upper smoke region, the flame region at the lower left of the smoke, and the flame region beneath the smoke but also detected the flame region embedded within the smoke plumes. Furthermore, the improved YOLO11 model produced significantly higher confidence scores for all detected smoke and flame targets in these scenes.

The qualitative visual detection results presented in Figure 6, Figure 7, Figure 8 and Figure 9 clearly demonstrate that the proposed improved YOLO11 model achieves a significant enhancement in detection performance relative to the baseline YOLO11 model across a diverse range of complex forest fire scenarios. The proposed improved algorithm not only mitigates the miss detection issue prevalent in complex scenarios but also enhances the model’s practical utility for real-world forest fire detection applications. These results collectively confirm the efficacy of the proposed improved algorithm for forest fire detection tasks, thereby verifying the rationality and feasibility of the integrated ShuffleNet-V1 and SPD-Conv module design.

## 4. Conclusions

A forest fire detection method based on a modified YOLO11 is proposed. In this model, the C3K2 modules are replaced with ShuffleNetV1 modules. The ShuffleNetV1 convolutional neural network incorporates grouped convolution and channel shuffle operations, which reduce the computational overhead of convolutional layers, preserve inter-channel information flow, minimize memory consumption, and ultimately achieve model lightweighting. Additionally, the strided convolutional layers and pooling layers in the YOLO11 model are replaced by SPD layers and non-strided convolutional layers, respectively. The information loss caused by downsampling is mitigated through the proposed SPD-Conv module, achieving richer feature fusion, thereby enhancing the extraction capability of forest fire features and improving the accuracy of small-target forest fire detection.

Compared to the baseline YOLO11, our method achieves a 7.7 M reduction in parameter count, a 0.3% increase in mAP@0.5, and a 2.2 GFLOP decrease in computational complexity. It can be concluded that the proposed model achieves simultaneous optimization in both model lightweighting and detection accuracy, and the efficacy and practical utility of the proposed method for real-world forest fire detection tasks are validated.

However, the proposed improved method still exhibits certain limitations. Specifically, it fails to accurately detect smoke and flame under extreme weather conditions (e.g., heavy rain or blizzard) or when thick smoke completely obscures all flames. Furthermore, owing to the absence of nighttime thermal imaging technology integration, the model is excessively dependent on visible spectrum data, potentially resulting in false or missed detections of nighttime fires. Consequently, in future work, we will refine the model in these regards, with the aim of enhancing its versatility while maintaining its lightweight architecture and detection accuracy.

## Figures and Tables

**Table 1:** Public datasets used.

Dataset Name	Data Description	Dataset Link
KMU Fire & Smoke	Includes flames, wildfire smoke, flame-like moving objects, etc.	https://cvpr.kmu.ac.kr/ (26 March 2025)
Wildfire Observers	Contains 2929 images of wildfires and smoke	http://wildfire.fesb.hr/ (26 March 2025)
Vmote	Contains 38 videos of forest fire smoke	http://www2.imse-cnm.csic.es/vmote/ (26 March 2025)
Fire and Smoke Detection	Includes 25 fire and smoke videos	http://signal.ee.bilkent.edu.tr/VisiFire/ (26 March 2025)

**Table 2:** Experimental platform configuration.

Category	Configuration
Operating System	Windows 11 CPU 2.40 GHz
CPU	Intel(R) Core(TM) i5-9300H CPU @ 2.40 GHz
GPU	NVIDIA GeForce GTX 1660 Ti
Programming Language	Python 3.8
Deep Learning Framework	PyTorch 2.5.1

**Table 3:** Ablation experiment of different modules in the improved model.

YOLO11	ShuffleNetv1	SPD-Conv	Parameters/M	GFLOPs	mAP@0.5 (%)
√			34.2	14.7	84.3
√	√		24.4	12.3	83.8
√		√	34.4	14.5	84.9
√	√	√	26.5	12.5	84.6

**Table 4:** Comparison of experimental results of different models.

Model	Parameters/M	GFLOPs	mAP@0.5 (%)	FPS
YOLOv8	37.9	18.4	83.6	115.3
YOLOv9	36.7	17.5	82.7	124.0
YOLOv10	34.4	17.3	82.2	148.6
YOLO11	34.2	14.7	84.3	164.6
Faster R-CNN	153.7	203.5	79.3	13.6
Ours	26.5	12.5	84.6	148.3

## Data Availability

The original contributions presented in this study are included in the article. Further inquiries can be directed to the corresponding author.
